# Changing the landscape of European School of Oncology–European Society for Medical Oncology masterclasses in clinical oncology during the COVID-19 pandemic

**DOI:** 10.2217/fon-2021-1477

**Published:** 2022-06-20

**Authors:** Nicholas Pavlidis, Fedro A Peccatori, Alexandru Eniu, Elie Rassy, Matti Aapro, Franco Cavalli, Florian Lordick, Alberto Costa

**Affiliations:** ^1^University of Ioannina, Ioannina, Greece; ^2^European School of Oncology College (ESCO), Milan, 20121, Italy; ^3^European School of Oncology (ESO), Milan, 20121, Italy; ^4^Gynecologic Oncology Department, European Institute of Oncology IRCCS, Milan, 20141, Italy; ^5^Hospital Riviera Chablais, Rennaz, 1847, Switzerland; ^6^Gustave Roussy, Département de Médecine Oncologique, Villejuif, 94805, France; ^7^Breast Center, Genolier Cancer Centre, Genolier, 1272, Switzerland; ^8^Oncology Institute of Southern Switzerland, Bellinzona, 6500, Switzerland; ^9^University of Leipzig Medical Centre, University Cancer Center (UCCL), Leipzig, 04103, Germany

**Keywords:** COVID-19, education, in-person, oncology, teaching, virtual

## Abstract

**Aims:** This study aimed to assess the participants' evaluation of the European School of Oncology–European Society for Medical Oncology virtual masterclasses in clinical oncology (MCOs) organized during the pandemic in 2021. **Materials & methods:** The participants answered an online evaluation questionnaire at the end of each MCO to evaluate the content and organization of the MCO. **Results:** The clinical session and case presentation scores ranged between 4.6 and 4.8 over 5. The participants strongly agreed that the MCOs offered updates to improve their knowledge and practice in 68–83% and 52–76%, respectively; 74–90% of the participants considered the quality of the meetings to be excellent. **Conclusion:** The participants were satisfied with the virtual MCOs during the COVID-19 pandemic. Virtual MCO may be an acceptable alternative educational modality in specific circumstances.

## Plain language summary

In 2002, the European School of Oncology (ESO) established masterclasses in clinical oncology (MCOs) and provided 41 in-person courses over the past two decades. As the COVID-19 pandemic forced travel restrictions and social distancing, the ESO and the European Society for Medical Oncology (ESMO) adapted the traditional MCOs to create virtual MCOs presented on e-ESO, an ESO e-learning platform. To date, five virtual MCOs have been organized for oncologists from western Europe, Latin America, Arab countries and southern Europe, the Baltic and Eurasia, eastern Europe and the Balkans. This study aimed to assess the participants' evaluation of the ESO-ESMO virtual MCOs organized during the pandemic in 2021 and to compare the participants' evaluation with that of previous in-person MCOs conducted between 2002 and 2019.

The far-reaching consequences of the COVID-19 pandemic have led to considerable changes in medical educational programs [1–4]. COVID-19 dismantled healthcare systems worldwide and led to significant anxiety and burnout among many oncologists [5]. As cases were growing worldwide, several healthcare systems redeployed oncologists, as well as the members of other specialties, to the frontline, thus disrupting training programs and face-to-face training [6,7]. The European Society for Medical Oncology (ESMO) has produced a collection of recommendations to guide the management of patients with cancer during the pandemic and supported the well-being of oncology professionals [8]. However, cancer education of young fellows and oncologists seemed to be overlooked during the pandemic, with many cancer educational events being canceled, interrupted or delayed [9].

The European School of Oncology (ESO) and ESMO masterclasses in clinical oncology (MCOs) are full, 5-day, interactive educational courses designed to enhance participants' knowledge and skills in medical oncology [10]. During an MCO, the ESO and ESMO faculty experts present in-person lectures that cover the latest diagnostic and treatment updates in oncology. Since 2002, ESO and ESMO have organized in-person MCOs in five different geographic regions yearly. The authors have previously reported the positive impact of ESO-ESMO MCOs on self-professional development, as 95% of the participants in MCOs considered this meeting useful for their professional career [11]. Before the pandemic, young oncologists attended the MCOs in person and commonly reported a high level of satisfaction with the goal, content and organization of the meetings [12,13]. Due to travel restrictions related to the COVID-19 pandemic, ESO canceled the MCO in 2020 and transitioned to a virtual environment in 2021 via e-ESO, an ESO e-learning platform. The authors conducted a survey to assess the participants' evaluation of the five ESO-ESMO virtual MCOs organized during the pandemic and compared the participants' evaluation with the previous in-person MCOs conducted between 2002 and 2019.

## Materials & methods

### Virtual MCO description

The virtual MCOs were teaching courses designed for medical and clinical oncologists to discuss patient management by cancer site and the latest updates. As in in-person meetings, eligible participants were selected on a competitive basis according to their curriculum vitae by a panel of ESO and ESMO experts. Participants and lecturers were emailed an access link prior to the scheduled lecture to provide instructions on navigating the online conferencing platform. The virtual MCO consisted of 13 hours of pre-recorded educational lectures and a 2-day live, online educational event that focused on specific topics of interest for oncologists. The pre-recorded lectures were made available to the participants 1 month before the live, online event. Participants attended these courses and answered three evaluation questions related to each session. During the live, online event, each lecture was followed by a question-and-answer (Q&A) session using some of the questions that were mentioned in the pre-recorded sessions. After each section, the panel of experts discussed the latest updates and solicited questions from the participants for approximately 10 min. Prior to the meeting, participants had to work in pairs to prepare clinical cases, which were presented and discussed with the faculty members during the live, online event [14]. ESO opted for this activity to increase networking among attendees, given that this opportunity is often missing from online educational activities. At the end of the MCO, participants were required to complete a self-assessment test [15].

### Evaluation of the MCOs

Participants were asked about their experiences with the virtual MCO using an online evaluation questionnaire that was addressed by an email invitation. Only one reminder email was sent thereafter; the reminder was not personalized and encouraged participants to answer the survey by offering the possibility to join the College of the European School of Oncology [16]. Responses were anonymous and could not be linked to the participant's score on the final self-assessment test.

A questionnaire of five sections was developed by the ESO faculty at the inception of the in-person MCO and was approved by the ESO ethics board [15]. The first section consisted of four demographic questions. In the second section, three items evaluated whether the MCO improved the participants' knowledge of and skills in the best clinical practice and ranked the quality of the education offered during the virtual MCO. The third and fourth sections covered the content and organization of the MCO in four and three items, respectively. The last section evaluated the presentations separately according to the slides' reliability, English quality and general feedback. Responses were collected using Likert scales: strongly agree, agree, neutral, disagree and strongly disagree or excellent, good, fair, poor and very poor. At the end of each section, freehand text answers were allowed to suggest areas of improvement.

## Results

Of the 281 participants in the five ESO-ESMO MCOs, 200 (71%) completed the survey 65% of the participants were female. The majority of the participants were medical or clinical oncologists (n = 119 [69.5%] and 59 [29.5%], respectively; Table 1). The participants were of 56 nationalities and originated mainly from Portugal (n = 22; 11%), the Russian Federation (n = 20; 10%), Italy (n = 15; 7.5%), Egypt (n = 14; 7%) and Mexico (n = 12; 6%; Table 2).

**Table 1.** Demographics of the selected participants in the masterclasses in clinical oncology.

	Western Europe	Latin America	Arab countries and southern Europe	Baltic and Eurasia	Eastern Europe and Balkans
**Participants (n)**	59	42	32	36	31
**Age**					
– <35 years	38 (64.4%)	27 (64.3%)	23 (71.9%)	32 (88.9%)	25 (80.6%)
– 35–45 years	18 (30.5%)	15 (35.7%)	9 (28.1%)	4 (11.1%)	6 (19.4%)
– >45 years	3 (5.1%)				
**Gender**					
– Female	46 (78.0%)	20 (47.6%)	24 (75.0%)	23 (63.9%)	22 (71.0%)
– Male	13 (22.0%)	22 (52.3%)	8 (25.0%)	13 (36.1%)	9 (29.0%)
**Specialty**					
– MO	37 (62.7%)	29 (69.0%)	11 (34.4%)	23 (63.9%)	19 (61.3%)
– CO	16 (27.1%)	13 (31.0%)	12 (37.5%)	10 (27.8%)	8 (25.8%)
– RO	0 (0%)	0 (0%)	8 (25.0%)	2 (5.6%)	3 (9.7%)
– HO	2 (3.4%)	0 (0%)	1 (3.1%)	0 (0%)	0 (0%)
– NM	2 (3.4%)	0 (0%)	0 (0%)	0 (0%)	0 (0%)
– Other	2 (3.4%)	0 (0%)	0 (0%)	1 (2.8%)	1 (3.2%)

CO: Clinical oncology; HO: Hematology oncology; MO: Medical oncology; NM: Nuclear medicine; RO: Radiotherapy oncology.

**Table 2.** Distribution of participants by country of origin.

Western Europe (n = 59)		Latin America (n = 42)		Arab countries and southern Europe (n = 32)		Baltic and Eurasia (n = 36)		Eastern Europe and Balkans (n = 31)	
Portugal	19 (32.2%)	Mexico	12 (28.6%)	Egypt	13 (40.6%)	Russian Federation	17 (47.2%)	Romania	7 (22.6%)
Italy	14 (23.7%)	Brazil	8 (19.0%)	Tunisia	5 (15.6%)	Ukraine	5 (13.8%)	Serbia	4 (12.9%)
Belgium	3 (5.1%)	Argentina	5 (11.9%)	Jordan	4 (12.5%)	Portugal	3 (8.3%)	Albania	3 (9.7%)
Sweden	3 (5.1%)	Chile	4 (9.5%)	Lebanon	2 (6.3%)	Azerbaijan	2 (5.6%)	Hungary	2 (6.5%)
United Kingdom	3 (5.1%)	Peru	4 (9.5%)	Albania	1 (3.1%)	Georgia	2 (5.6%)	Bulgaria	2 (6.5%)
Austria	2 (3.4%)	Costa Rica	3 (7.1%)	Iraq	1 (3.1%)	Armenia	1 (2.7%)	Russian Federation	2 (6.5%)
France	2 (3.4%)	Cuba	2 (4.7%)	Ireland	1 (3.1%)	Belarus	1 (2.7%)	Republic of North Macedonia	2 (6.5%)
Ireland	2 (3.4%)	Bolivia	1 (2.3%)	Libya	1 (3.1%)	China	1 (2.7%)	Croatia	1 (3.2%)
Spain	2 (3.4%)	Colombia	1 (2.3%)	Morocco	1 (3.1%)	Latvia	1 (2.7%)	France	1 (3.2%)
Finland	1 (1.7%)	El Salvador	1 (2.3%)	Russian Federation	1 (3.1%)	Romania	1 (2.7%)	Iran	1 (3.2%)
Greece	1 (1.7%)	Paraguay	1 (2.3%)	Saudi Arabia	1 (3.1%)	Turkey	1 (2.7%)	Italy	1 (3.2%)
Romania	1 (1.7%)			Turkey	1 (3.1%)	Uzbekistan	1 (2.7%)	Poland	1 (3.2%)
Switzerland	1 (1.7%)							Slovak Republic	1 (3.2%)
Egypt	1 (1.7%)							Slovenia	1 (3.2%)
Lebanon	1 (1.7%)							Turkey	1 (3.2%)
Morocco	1 (1.7%)								
South Africa	1 (1.7%)								
Hong Kong	1 (1.7%)								
Australia	1 (1.7%)								

Regarding the evaluation scores of the clinical sessions and clinical case presentations, the overall scores ranged between 4.6 and 4.8 over 5 in the different geographic regions; the lowest score was in the Arab countries and southern Europe MCO (Table 3). Most of the clinical sessions evaluated by the participants, as well as the clinical case presentations presented by the participants, were highly valued, with an overall scoring of 4.5–4.9 and 4.7–4.9, respectively.

**Table 3.** Evaluation scores^†^ of the clinical sessions and clinical case presentations by region.

Clinical session/case presentation	Western Europe	Latin America	Arab countries and southern Europe	Baltic and Eurasia	Eastern Europe and Balkans
Breast cancer	4.77	4.85	4.66	4.79	4.75
Clinical case discussion	4.70	4.86	4.70	4.82	4.79
Lung cancer	4.70	4.75	4.54	4.75	4.14
Clinical case discussion	4.77	4.89	4.58	4.84	4.86
Gastrointestinal cancers	4.76	4.84	4.57	4.76	4.14
Clinical case discussion	4.78	4.90	4.71	4.85	4.78
Gynecological cancers	4.76	4.82	4.57	4.81	4.72
Clinical case discussion	4.83	4.84	4.67	NA	4.90
Genitourinary cancers	4.75	4.79	4.55	4.78	4.74
Clinical case discussion	4.81	NA	NA	NA	4.88
Spotlights	4.62	4.62	4.50	4.77	4.69
Overall	4.75	4.81	4.60	4.79	4.67

† Scores ranged between 1 and 5, with 5 being the highest evaluation.

NA: Not applicable.

The participants strongly agreed that the MCO offered updates to improve their knowledge in the field and practice in 68–83% and 52–76%, respectively; 74–90% of the participants considered the quality of the meeting to be excellent. The Arab countries and southern Europe MCO participants reported the lowest satisfaction score (Table 4). Among the participants in MCOs, the content of the sessions was well balanced and supported with evidence in 58–77% and the time of the discussion sessions was satisfying in 52–68%. In the evaluation of the event organization, 81–89% of the participants considered the organization and management of the meeting to be excellent, albeit a lower satisfaction with the platform, ranging between 68 and 84%.

**Table 4.** Proportions of participants who responded ‘strongly agree’ or ‘excellent’ in the general, content and organization evaluation sections of the survey.

	Western Europe	Latin America	Arab countries and Southern Europe	Baltic and Eurasia	Eastern European and Balkans
Did the e-course offer qualified updates?	83%	83%	68%	71%	77%
Did the e-course improve your skills and your practice?	71%	76%	52%	54%	68%
Give an overall rating for the quality of the e-course.	85%	90%	74%	80%	90%
Was the program well balanced and supported with evidence?	73%	83%	58%	71%	77%
Did the program allow adequate time for discussion?	51%	52%	68%	63%	61%
How would you rate the organization and management of the meeting?	85%	88%	81%	89%	87%
How would you rate the technical system of this e-course?	71%	76%	68%	74%	84%

The Supplementary Material details the answers provided in the freehand text items intended to suggest areas of improvement. Overall, participants were satisfied with the content and organization. The main suggested area of improvement was relevant to the time of interaction for discussion and questions between the faculty and the participants.

## Discussion

The COVID-19 pandemic led to an unprecedented cancelation of the ESO-ESMO MCOs during 2020, rather than an abrupt transition to a virtual teaching environment to deliver didactic lectures during an exhausting period. Nevertheless, the authors primary objectives were in line with the Accreditation Council for Graduate Medical Education (ACGME) and considered that training and teaching programs must continue to provide adequate resources, training and supervision [17]. As previously mentioned, ESMO had already published a series of guidelines for the management of patients during the pandemic. Thereafter, ESO designed e-ESO, a dedicated platform suitable for the MCO lectures and interactions.

This survey aimed to assess the first virtual MCO experience for participants by assessing their satisfaction in regard to the goals, content and organization of the masterclasses. Overall, the results reflect that the majority of the participants were satisfied with the MCOs despite the transition from the traditional in-person to virtual lectures. Interestingly, the item relevant to discussion time seemed to score the lowest, which may reflect the challenging part of virtual meetings with large audiences. Indeed, many participants suggested increasing the duration of the interaction with the experts in order to improve the quality of the virtual MCOs. Moreover, participants commonly discuss their queries with the experts after their presentations during in-person events, whereas this accessibility to the experts is not possible during a virtual MCO. The authors attempted to understand the participants' satisfaction according to their geographic region and detected that the Arab countries and southern Europe MCO had the lowest score for the majority of the items relevant to the goals, content and organization of the meeting. Notably, participants in the Arab countries and southern Europe MCO had the highest proportion of satisfaction with the time dedicated for discussions with the expert panel. This finding underlines a differential need for information among the geographic regions [18–20].

**Figure 1.**
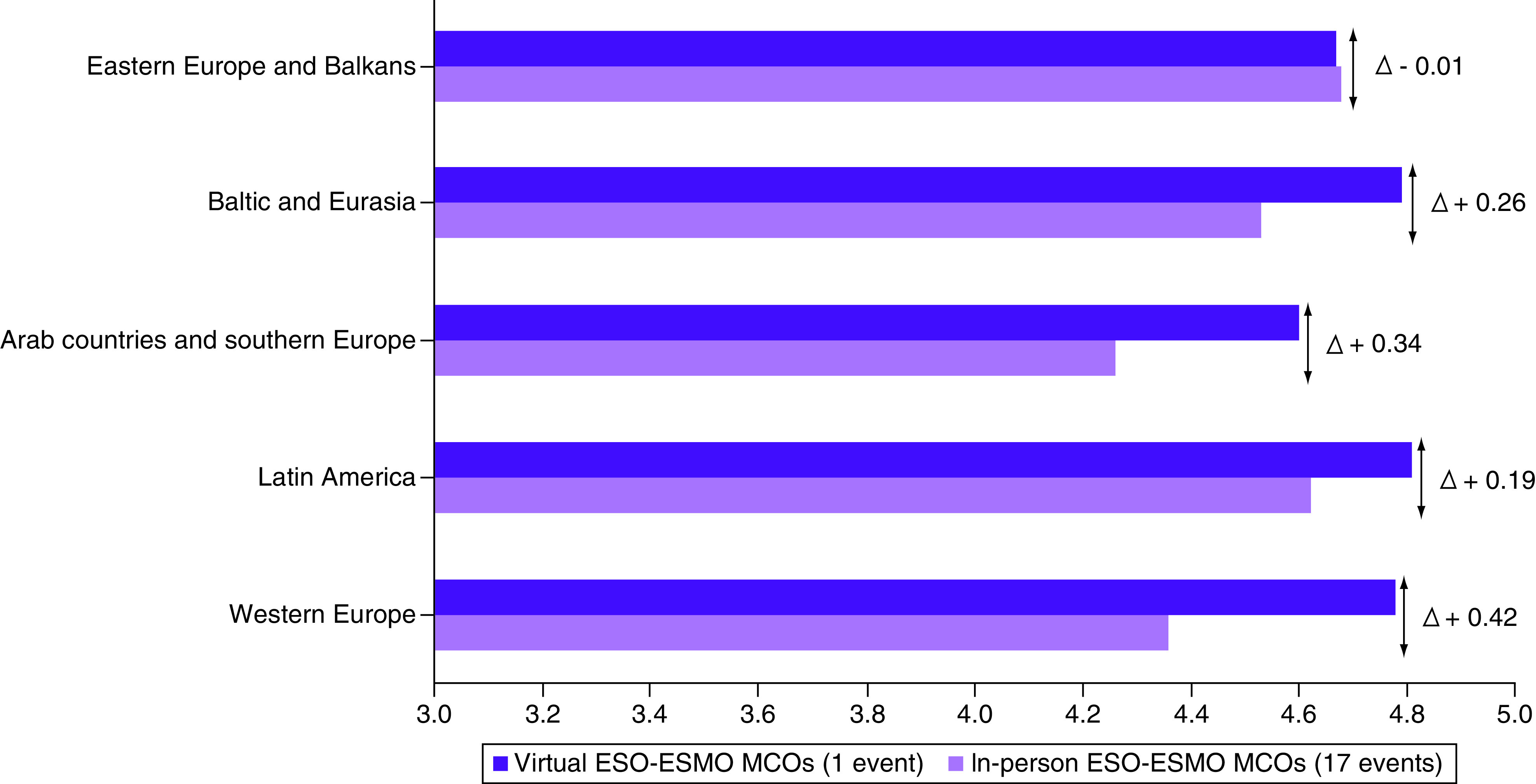
Evaluation scores of the virtual European School of Oncology–European Society for Medical Oncology masterclasses in clinical oncology and the previous 17 in-person masterclasses in clinical oncology. ESO-ESMO: European School of Oncology–European Society for Medical Oncology; MCO: Masterclasses in clinical oncology.

Previous publications have shown that virtual teaching has not been favored over the traditional in-person approach [21–24]. However, these findings cannot be extrapolated to the medical education during the COVID-19 pandemic period, as the workforce was directed toward patient care and teaching activities were suspended. A recent publication reporting on the online education of oncology fellows during the pandemic has shown that most participants felt comfortable with the transition to a fully virtual didactic curriculum [25]. The MCOs occurred during a period with a growing optimism regarding the effectiveness of the COVID-19 vaccine in preventing severe infection and a trend toward involving, at least partly, in-person meetings. Nevertheless, the authors' experience showed that the virtual ESO-ESMO MCOs achieved higher satisfaction scores in comparison with historical controls from the previous 17 in-person MCOs across the five geographic regions (Figure 1). The survey did not detail the parameters that would influence such improvement because MCOs were originally designed to favor networking and social engagement. The higher differential satisfaction improvements in the western Europe MCOs followed by the Arab countries and southern Europe MCOs may be attributed to the COVID-19 pandemic surges and cost/convenience benefits, respectively. A satisfaction survey among radiation oncology trainees showed that the reduced cost and travel time were the most common reasons to favor virtual meetings, especially among those training in non-metropolitan centers [26].

Although the current study may be generalizable to the different geographic regions involved, several limitations should be acknowledged. The number of participants in the virtual MCOs was similar to that in the in-person MCOs; nevertheless, the sample size considered for this analysis was quite small. Unfortunately, the authors did not address the survey to the oncologists who had registered for the course but dropped out; however, the concordance of the dropout rate with those of previous years did not allude to particular issues. Finally, this survey may have favored participants with easy access to electronic tools. Participants were not asked specifically about the challenges of the participation modalities, but two issues can be raised. Many participants complained about the short duration of the interaction with the experts and showed lower satisfaction concerning this item of the survey. A minority of the participants found the e-ESO platform complex but quickly adapted to its tools.

## Conclusion & future perspective

The COVID-19 pandemic has accelerated the embrace of modern technologies in academic interactions to maintain continuous medical education, especially during difficult times when expert advice is required. The results of this survey demonstrate that virtual MCOs can be amenable to incorporation into oncology education. This positive feedback from participants from five different geographic regions supports the acceptability of the virtual approach in delivering medical oncology updates. Furthermore, the results of the survey are instructive in directing ESO-ESMO faculty members to adapt the virtual sessions according to the participants' needs.

**Summary points**

**Introduction**

- This study aimed to assess the participants' evaluation of the European School of Oncology–European Society for Medical Oncology virtual masterclasses in clinical oncology (MCOs) organized during the pandemic in 2021.

**Methods**

- Participants answered an online evaluation questionnaire at the end of each MCO to evaluate the content and organization of the MCO.

**Results**

- Participants were highly satisfied with virtual MCOs during the COVID-19 pandemic.
- The majority of the participants considered the quality of the meetings to be excellent.
- The Arab countries and southern Europe virtual MCOs had the lowest evaluation.
- The western Europe virtual MCOs had higher improvement in evaluation compared with the in-person events.
- Participants complained mainly of the duration of the interaction with the experts.

**Conclusion**

- Virtual MCOs may be an acceptable alternative educational modality in specific circumstances.
